# Exceptional lability of a genomic complex in rice and its close relatives revealed by interspecific and intraspecific comparison and population analysis

**DOI:** 10.1186/1471-2164-12-142

**Published:** 2011-03-08

**Authors:** Zhixi Tian, Yanjun Yu, Feng Lin, Yeisoo Yu, Phillip J SanMiguel, Rod A Wing, Susan R McCouch, Jianxin Ma, Scott A Jackson

**Affiliations:** 1 Department of Agronomy, Purdue University, West Lafayette, IN 47907, USA; 2 Arizona Genomics Institute, The University of Arizona, Tucson, AZ 85721, USA; 3 Genomics Core Facility, Purdue University, West Lafayette, IN 47907, USA; 4 Department of Plant breeding and Genetics, Cornell University, NY 14853, USA; 5 Department of Biological Sciences, Purdue University, West Lafayette, IN 47907, USA

## Abstract

**Background:**

Extensive DNA rearrangement of genic colinearity, as revealed by comparison of orthologous genomic regions, has been shown to be a general concept describing evolutionary dynamics of plant genomes. However, the nature, timing, lineages and adaptation of local genomic rearrangement in closely related species (*e.g*., within a genus) and haplotype variation of genomic rearrangement within populations have not been well documented.

**Results:**

We previously identified a hotspot for genic rearrangement and transposon accumulation in the *Orp *region of Asian rice (*Oryza sativa*, AA) by comparison with its orthologous region in sorghum. Here, we report the comparative analysis of this region with its orthologous regions in the wild progenitor species (*O. nivara*, AA) of Asian rice and African rice (*O. glaberrima*) using the BB genome *Oryza *species (*O. punctata*) as an outgroup, and investigation of transposon insertion sites and a segmental inversion event in the AA genomes at the population level. We found that *Orp *region was primarily and recently expanded in the Asian rice species *O. sativa *and *O. nivara*. LTR-retrotransposons shared by the three AA-genomic regions have been fixed in all the 94 varieties that represent different populations of the AA-genome species/subspecies, indicating their adaptive role in genome differentiation. However, LTR-retrotransposons unique to either *O. nivara *or *O. sativa *regions exhibited dramatic haplotype variation regarding their presence or absence between or within populations/subpopulations.

**Conclusions:**

The LTR-retrotransposon insertion hotspot in the *Orp *region was formed recently, independently and concurrently in different AA-genome species, and that the genic rearrangements detected in different species appear to be differentially triggered by transposable elements. This region is located near the end of the short arm of chromosome 8 and contains a high proportion of LTR-retrotransposons similar to observed in the centromeric region of this same chromosome, and thus may represent a genomic region that has recently switched from euchromatic to heterochromatic states. The haplotype variation of LTR-retrotransposon insertions within this region reveals substantial admixture among various subpopulations as established by molecular markers at the whole genome level, and can be used to develop retrotransposon junction markers for simple and rapid classification of *O. sativa *germplasm.

## Background

Comparative genetic mapping and comparison of orthologous genomic sequences of grasses, such as rice, maize, sorghum, barley, wheat, and millet have demonstrated extensive genomic colinearity among species that radiated from common ancestors ~10-60 million years ago [1,2]. Although numerous and different levels of genomic rearrangements, including gene movement, and loss or creation of new genes were uncovered in some grass lineages [3-5], gene content has been shown to be highly conserved between species. For instance, all genes, including single-copy ones, absent in the genomic region surrounding the orange pericarp (*Orp*) gene of rice, in contrast to its orthologous regions of sorghum and maize, were found elsewhere in the rice genome and even in *Arabidopsis *[3,6]. Comparison of homoeologous segments of maize revealed exceptionally high-level of loss of one of the homoeologous gene pairs [3,5-7], which appears to be a general phenomenon in the evolution of any polyploid organism toward a diploid genomic state. These dynamic processes of gene duplication and deletion may explain why rice and *Arabidopsis *share a similar set of genes, although their genomes have undergone separate paleopolyploidy and/or segmental duplication events during their ~120 million year independent evolution [8].

In contrast to genes, intergenic spaces were found to be less or not conserved between grasses, such as maize and sorghum. Intergenic sequences are generally composed of transposable elements or transposable element fragments, primarily long terminal repeat (LTR)-retrotransposons, and other unknown DNA components. Given that most structurally detectable LTR-retrotransposons were amplified within last a few million years [9,10], it is not surprising that substantial differences in intergenic regions have been found between subspecies of rice [11], or even between inbreds of maize [12,13]. On the other hand, LTR-retrotransposons can be partially or completely deleted from the host genomes within very short evolutionary timeframes. For example, it was estimated that ~200 Mb of LTR-retrotransposon DNA was removed from the rice genome by unequal homologous recombination and illegitimate recombination within the past 5 million years [9,10], although neither amplification nor removal of LTR-retrotransposons seems to be absolutely gradual processes [14]. In addition to the gain and loss of transposable elements, intergenic sequences generally diverge more rapidly than genic sequences by nucleotide substitution [10,15]. These dynamic processes have led to the scarcity of conserved intergenic sequences, even between moderately diverged grass lineages such as maize and sorghum [6,16].

Comparison of closely related species, subspecies, and/or different haplotypes or ecotypes is a promising approach to investigate more recent evolutionary events. A comparative sequence analysis of ~1.1 Mb orthologous regions of two subspecies of rice, *indica *and *japonica*, revealed more than 2% and 6% growth of two respective genomes over the past half million years, primarily by amplification of LTR-retrotransposons [10]. Wang and Dooner presented a comprehensive comparison of seven inbred lines of maize, demonstrating the remarkable haplotype variation of the *bz *genomic regions caused predominantly by insertion of LTR-retrotransposons, helitrons, DNA transposons and other new repetitive components [17]. However, the dynamic variation of transposable elements, their potential interplay with genic rearrangement, and their roles for genomic selection and diversity remain to be investigated, particularly, at the population level.

The high-quality genomic sequence of rice [18] and genomic resources (e.g., BAC libraries, BAC end sequences, BAC-based physical maps) generated by the ongoing *Oryza *map alignment project (OMAP) [19] provide an unprecedented opportunity for research community to study the evolution of plant genomes within a genus. To date, three genomic (*Adh1*, *MOC1*, and *Hd1*) regions of multiple *Oryza *species have been investigated [20-22]. Because the *Oryza *species included in OMAP span evolutionary scales from < 1 million years to ~15 million years, as indicated by their phylogeny [23], comparisons of multiple *Oryza *species in these regions have uncovered some specific evolutionary events in specific lineages during the radiation of the *Oryza *species. However, all three regions are gene-rich and repeat-poor, therefore little is known about how transposable elements have affected the instability of the *Oryza *genomes during their speciation and diversification.

A hotspot of transposable element accumulation that harbors a few truncated and duplicated gene fragments was previously described between two gene clusters of the *Orp *region of rice (*O. sativa *ssp. *japonica*). This hotspot is located near the end of the short arm of chromosome 8 (from 1757 to 1997 kb, rice Pseudomolecule 4.0), and contains a high proportion of LTR-retrotransposons, similar to that observed in the centromeric region (Cen8) of this same chromosome [6,24], but it is absent in the corresponding regions of sorghum and maize [6]. To track the evolutionary history of the formation of this hotspot and the spectrum of genic rearrangements involved, we identified its orthologous regions from AA-, BB-, EE-, and FF-genome *Oryza *species by searching the *O. sativa Orp *region against BAC end sequences (BESs) generated by OMAP. In particular, we sequenced two overlapping BAC clones from *O. nivara *(AA), one of the proposed wild progenitors of Asian rice (*O. sativa*), one BAC clone from *O. glaberrima *(AA), the cultivated rice species domesticated in African, and one BAC clone from *O. punctata *(BB). In addition, we investigated the haplotype variation of LTR-retrotransposon insertions and an inversion of a genomic segment within the hotspot. We present here the comparative genomic analysis of these orthologous regions and haplotype variation mediated by LTR-retrotransposons, thereby depicting the nature, timing, rate and specificity of DNA changes observed in these regions during the speciation and diversification of these closely related *Oryza *species.

## Results and Discussion

### Size variation of the *Orp *orthologous regions across *Oryza *species reflects recent genomic expansion

**Figure 1:**
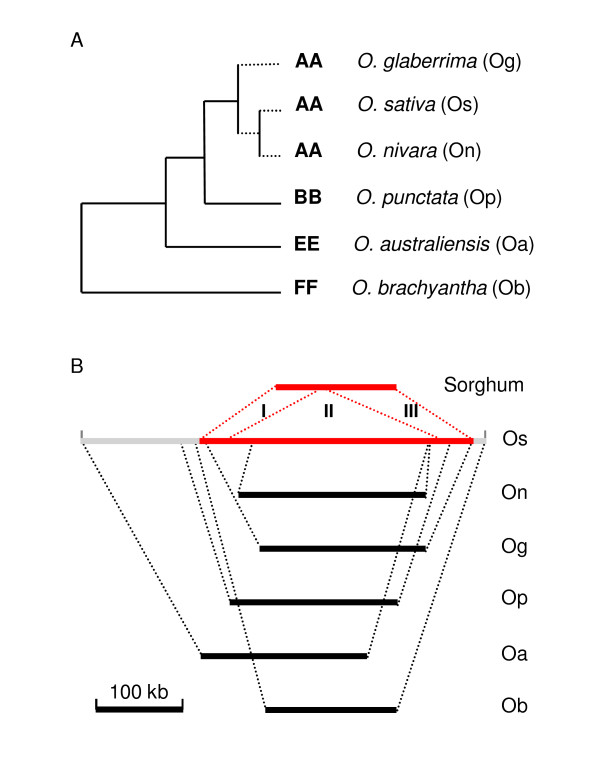
**Size variation of the orthologous *Orp *regions of *Oryza *species**. (A) Phylogeny of the *Oryza *species [adapted from [23]]. (B) Contraction/expansion of the *Orp *regions in *O. nivara*, *O. glaberrima*, *O. punctata*, *O. australiensis*, and *O. brachyantha *relative to the orthologous region in *O. sativa*. Dotted red lines connecting the *O. sativa *and sorghum regions mark two gene clusters interrupted by a hotspot of transposon insertions and genic rearrangements in *O. sativa *as previously described by Ma et al. 2005. Dotted dark lines mark the boundaries of the orthologous regions defined by anchoring BESs from other *Oryza *species to the *O. sativa *sequence of the *Orp *region.

To select the genomic segments that are orthologous to the *O. sativa Orp *regions in other *Oryza *species, we searched a set of BAC end sequences (BESs) derived from the *O. nivara*, *O. glaberrima*, *O. punctata*, *O. australiensis*, and *O. brachyantha *genomes (OMAP, http://www.omap.org, Figure 1A). Individual BAC clones with two BESs anchored to the unique sequences of the *O. sativa Orp *region and/or its flanking regions in opposite orientations were considered to be orthologous segments. One or two overlapping BAC clones from each species that maximally cover the hotspot of insertions of transposable elements in the *Oryza Orp *region [6] were chosen for sequencing and/or further analysis. BAC clones OR_BBa0014L06 from *O. nivara*, two overlapping clones OG_BBa0075G14 and OGBBa0001L21 from *O. glaberrima*, and clone OP__Ba0008J05 from *O. punctata *were completely sequenced. These sequenced genomic segments are 214 kb, 190 kb, and 192 kb in *O. nivara*, *O. glaberrima*, and *O. punctata*, respectively, corresponding to 202 kb, 309 kb, and 339 kb of the orthologous regions in *O. sativa *(Table 1). Thus, a 12-kb expansion of the *O. nivara *region and 119-kb and 147-kb contractions of the *O. glaberrima *and *O. punctata *regions relative to the corresponding orthologous regions in *O. sativa *were revealed. In addition, 212-kb and 192-kb contractions of the *O. australiensis *and *O. brachyantha *regions relative to the corresponding *O. sativa *orthologous regions (Table 1) were suggested by fingerprinting of BAC clones OA_ABa0108F22 and OB__Ba0050L03 (OMAP, http://www.omap.org) that define the orthologous *Orp *regions in *O. australiensis *and *O. brachyantha*, respectively (Figure 1B). The relative contraction of the *Orp *region in sorghum in contrast to the *Orp *region in *O. sativa *is 175 kb (Table 1), and no transposable elements were identified in the sorghum region [6]. These observations, together with the evolutionary relationship of these species as illustrated in Figure 1A, suggest that the *Orp *region was primarily and recently expanded in the Asian rice species *O. sativa *and *O. nivara *[i.e., after the divergence of Asian and African rice approximately 1.2 million years ago [25]]. Because this study aimed to decipher the nature and timing of recent genic rearrangements and gain and loss of LTR-retrotransposon in the *Orp *regions of the AA genome species, the orthologous BAC clones from *O. australiensis *and *O. brachyantha *were not further investigated.

**Table 1:** Size variation of the orthologous *Orp *regions of O*ryza *investigated

	Genome	Genome		Size of BAC or contig	Size of corresponding region in	Size variation relative to *O. sativa *(kb)	
						
Species^a^	designation	size^b ^(Mb)	BAC or contig	(kb)^c^	*O. sativa *(kb)^d^	Contraction	Expansion
*O. nivara*	AA	448	OR_BBa0014L06	214	202		12
*O. glaberrima*	AA	354	OG_BBa0075G14/OG_BBa0001L21	190	309	119	
*O. punctata*	BB	423	OP__Ba0008J05	192	339	147	
*O. australiensis*	EE	960	OA_Aba0108F22	190	401	212	
*O. brachyantha*	FF	338	OB__Ba0050L03	150	342	192	
Sorghum	HH	735	SB18C08	138^e^	313^e^	175	

^a^*Oryza *species refers to Wing et al., 2005 and sorghum refers to Ma et al., 2005

^b^Refers to Bennett and Leitch, 2003

^c^Estimated by gel electrophoreses of restriction enzyme-digested fragments or sequencing

^d^*O. sativa *subspecies, *japonica *(c.v., Nipponbare)

^e^The corresponding region comparable

### Sequence organization and comparison of the orthologous regions

**Figure 2:**
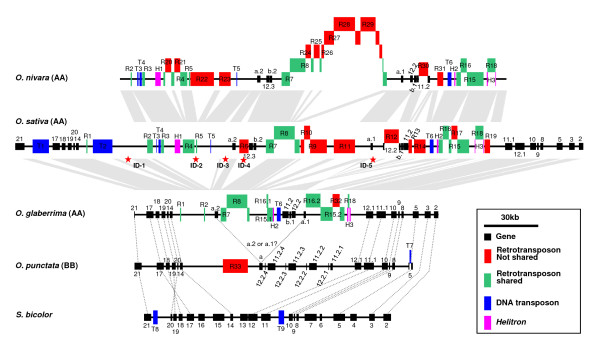
**Sequence organization and comparison of the *Orp *regions of *Oryza *species**. Solid dark box represent annotated genes or gene fragments. Colored boxed represent identified transposable elements. Dotted lines indicate orthologous genes. Grey blocks indicate orthologous segments in the *Orp *regions shared between two species compared. Stars indicate indels detected between *O. sativa *and *O. glaberrima*.

Analysis of the *Orp *orthologous regions in *O. sativa *and sorghum was previously performed [6]. Using these same criteria, we annotated the orthologous regions from *O. nivara*, *O. glaberrima*, and *O. punctata*. Sequence organizations of the five orthologous regions are illustrated in Figure 2 and detailed in Additional file 1, Table S1.

The 214-kb *O. nivara *region is comprised of 21 LTR-retrotransposons, 3 DNA transposons, 3 Helitrons, and 6 genes/pseudogenes. LTR-retrotransposons alone make up 121 kb of DNA sequence, accounting for 56.6% of the region. Although the *O. nivara *region is only 12 kb larger than its corresponding orthologous region in *O. sativa*, it was found that 12 LTR-retrotransposons (74 kb of DNA) in the former and 9 LTR-retrotransposons (55 kb of DNA) in the latter, were not shared in the two regions. Each of these unshared elements is flanked by 5-bp target site duplication in its host region with a single copy of the 5-bp "target site" in the other orthologous region, suggesting that these elements inserted into the current positions after the divergence of the two regions from their ancestral form. By contrast, all of the 3 DNA transposons and 3 *Helitrons *are shared in the two regions, suggesting that they were integrated before the divergence of the two regions. Overall, these two regions exhibit perfect colinearity in gene order and orientation, although gene 11.2 in *O. nivara *and gene 12.2 in *O. sativa *each had a LTR-retrotransposon insertion (Figure 2).

The 190-kb *O. glaberrima *region is comprised of 7 LTR-retrotransposons, 1 DNA transposon and 2 Helitrons. Nine out of these 10 transposable elements are shared by *O. glaberrima *and *O. sativa*, indicating that the *O. glaberrima *region did not expand substantially by the amplification of LTR-retrotransposons after its divergence from the *O. sativa *lineage. In addition to indels (insertions/deletions) generated by insertions of 2 DNA transposons (T1 and T2) and 8 LTR-retrotransposons (R9, R10, R11, R12, R13, R14, R17, and R18), and by the formation of a solo LTR (R2) through unequal recombination [26] in *O. glaberrima*, 5 relatively large indels (> 3 kb), ID-1, ID-2, ID-3, ID-4 and ID-5 (Figure 2) were observed between the two regions. Among these 5 indels, ID-2 is the largest (37 kb) and harbors 3 DNA transposons, 1 *Helitron*, and 3 LTR-retrotransposons present in *O. sativa*. Genes 12.3 and b.2 involved in ID-4 were present in *O. sativa *but absent in *O. glaberrima*. ID-5 appears to be a (4.3 kb) deletion that led to partial truncation of gene a1 and removal of a DNA transposon (4.3 kb) fragment in *O. glaberrima*. This deletion flanks the inverted segment in the *O. glaberrima *region that harbors genes 11.2, b1 and 12.2. The other breakpoint for this inversion is located within LTR-retrotransposon R16 (belonging to family *Osr14 *[27,28]) and as a result R16 was separated into two fragments (R16.1 and R16.2). Interestingly, a deletion of ~4.3 kb internal sequence of R16 at this breakpoint was deduced by comparing R16.1 and R16.2 with typical intact elements of the *Osr14 *family [27,28]. It is unclear whether the inversion led to the two flanking deletions or the latter caused the former. The *O. glaberrima *and *O. sativa *regions show overall colinearity except for the genic rearrangements described above.

The 148-kb *O. punctata *region has a single LTR-retrotransposon (R33), which is not shared by the three A-genome species. Based on the divergence of two LTRs of R33, it was estimated that this element was integrated into the region ~0.038 mya. This region shares perfect colinearity at the two gene clusters (i.e., genes 5, 8, 9, 10, 11, 12, and genes 14, 20, 19, 18, 17, 21) with the A-genome species, with the exception of a recent quaduplication of a segment containing two gene fragments (genes 11.2 and 12.2), which resulted in a substantial size increase of the interval between the two gene clusters in *O. punctata*.

### The nature and history of genic rearrangements

**Figure 3:**
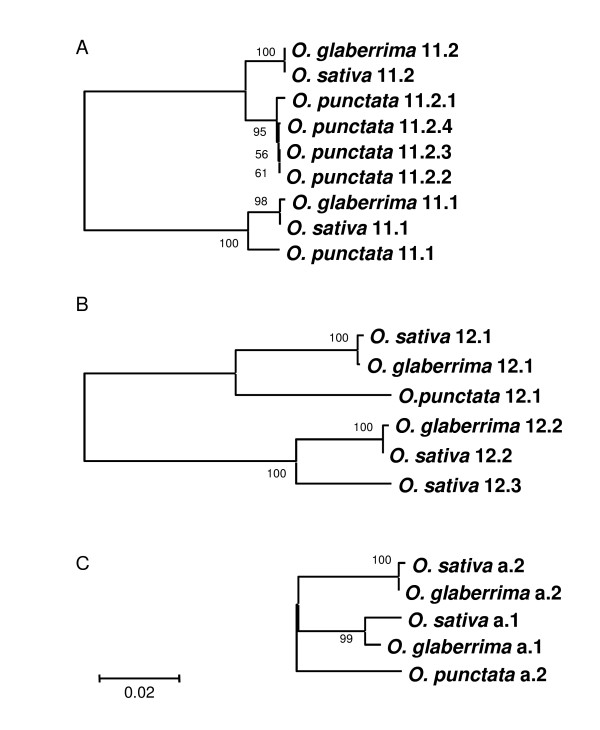
**Phylogenetic relationships of duplicated genes within and across species**. The phylogenetic tree was constructed based on nucleotide sequences of individual genes.

Most duplicated genes interspersed in the intervals of the two highly conserved gene clusters are pseudogenes or gene fragments, in which the protein-coding sequences cannot be accurately predicted. Thus, the gene duplication events observed in this study could not be dated based on protein-coding sequences. To illuminate the history of the duplication events, we performed phylogenetic analysis of the duplicated genes within and across species using their genomic sequences (Figure 3). As shown in Figure 3A, gene 11.1 and gene 11.2 from the three species, *O. sativa*, *O. glaberrima*, and *O. punctata*, were grouped into two distinct branches (i.e., gene 11.1 branch and gene 11.2 branch), and the phylogeny reflected by either branch is consistent with the evolutionary relationship among the three species [23]. These data suggest that gene 11.1 and gene 11.2 were duplicated before the divergence of AA and BB genome species from a common ancestor. Gene 12.2 (12.2.1, 12.2.2, 12.2.3 and 12.2.4) in *O. punctata *was largely truncated, and thus excluded in the phylogenetic analysis. Gene 12.1 in the three species shows a similar phylogenetic pattern (Figure 3B) as revealed by gene 11.1 and 11.2, suggesting that the duplication of gene 12.1 and 12.2 also occurred before the divergence of the AA and BB genomes. The genetic distances between genes 11.1 and 11.2 and between genes 12.1 and 12.2 differ (Figure 3A and 3B), but the genes 11.1 and 12.1 and genes 11.2 and 12.2 were found to be arranged in the same order and orientation in the duplicated fragments. Thus, it is most likely that the duplication of both genes was caused by a single event.

Genes 12.2 and 12.3 in *O. sativa *grouped in the same branch, distinct to the branch of gene 12.1, suggesting that the duplication of 12.2 and 12.3 occurred after the first duplication event that predates the divergence of the AA and BB genomes. Gene b.1 is present in the AA genome species, but absent in the BB genome and sorghum. If the conserved segments containing genes 11.1 and 12.1 are ancestral copies of genes 11 and 12, the insertion of gene b (b.1 or b.2) must have occurred after the first duplication event. Because the three genes in each of the two gene clusters (a.2, 12.3 and b.2 cluster, and a.1, 12.2, and b.1 cluster) are arranged in the same order and orientation, it is likely that these three genes were duplicated by a single event before the divergence of the Asian and African AA genomes. The levels of sequence divergences between genes 12.2 and 12.3 and between genes a.1 and a.2 in *O. sativa *are similar (Figure 3B and 3C), reinforcing this conclusion. Assuming this deduction is correct, the absence of genes 12.3 and b.2 in *O. glaberrima *must be the outcome of deletion(s) at ID-4 site (Figure 2).

Phylogenetic analysis revealed that gene a (a.1 or a.2) in *O. punctata *is nearly equally distinct to genes a.1 and a.2 in either AA genome species (Figure 3C), suggesting that the duplication of the gene a (i.e., a.1 and a.2) occurred near the split of the AA and BB genome species. Thus, the orthologous copy/copies of genes a between the AA and BB genomes cannot be deduced based on their sequence similarities. Phylogenetic analysis indicates that the four recently amplified copies of gene 11.2 in *O. punctata *are orthologous to gene 11.2 in *O. sativa *and *O. nivara *(Figure 3A). In comparison with the proposed two orthologous regions in the AA genomes, genes 12.3, b.2, b.1 and a.1 were absent in the BB genome (Figure 2).

**Figure 4:**
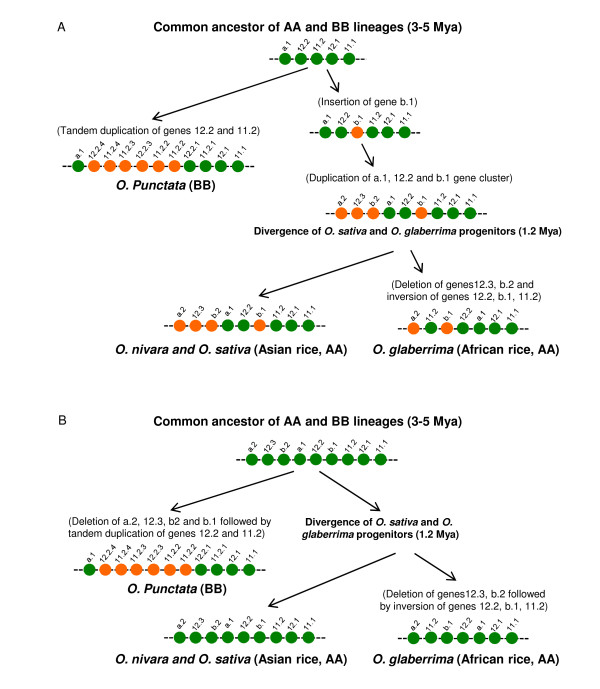
**Nature and evolutionary history of genic rearrangements in the *Orp *regions of *Oryza *species**.

According to the analyses above, we propose two evolutionary scenarios regarding genic arrangements and rearrangements in the *Oryza Orp *regions. The first scenario, as illustrated in Figure 4A, proposes that the initial copy of gene a (i.e., a.1) inserted before the divergence of the AA and BB genome, and the initial copy of gene b (i.e., b.1) inserted only in the AA species after the AA and BB species divergence. The duplication event was followed by the duplication of the gene cluster (a.1, 12.2 and b.1) that generated genes a.2, 12.3, and b.2 in the AA species. Based on this hypothesis, the absence of genes 12.3, b.2, b.1 and a.1 in the BB genome can be explained solely by "gain" of these genes in the *Orp *regions of the AA species. Of course, it is also possible that the insertions of initial copies of genes a and b and the subsequent duplication of the gene cluster (a.1, 12.2 and b.1) occurred before the divergence of the AA and BB species (Figure 4B). In this scenario case, the absence of genes seen in the BB genome could be explained by multiple gene deletion events, which is less parsimonious than the first scenario.

Regardless, our data revealed unusual structural instability in the *Oryza Orp *regions, including recent and rapid accumulation of LTR-retrotransposons and recent genic rearrangements. These genomic changes took place within an originally gene-rich euchromatic chromosome arm, reflecting a general plasticity of the *Oryza *genomes under the umbrella of local genic colinearity. Given that the structural variation of genomic regions can substantially affect chromatin states [29], frequencies of local recombination [13], and the expression/functionality of genes within or flanking the regions [30], the genomic plasticity revealed in this region, and probably many other genomic regions, as a general pattern, may have played a significant role, as proposed by Ginzburg et al. [31], in the processes of *Oryza *genome speciation.

### Population analysis of haplotype variation of LTR-retrotransposon insertions and segmental inversion in the AA species

**Figure 5:**
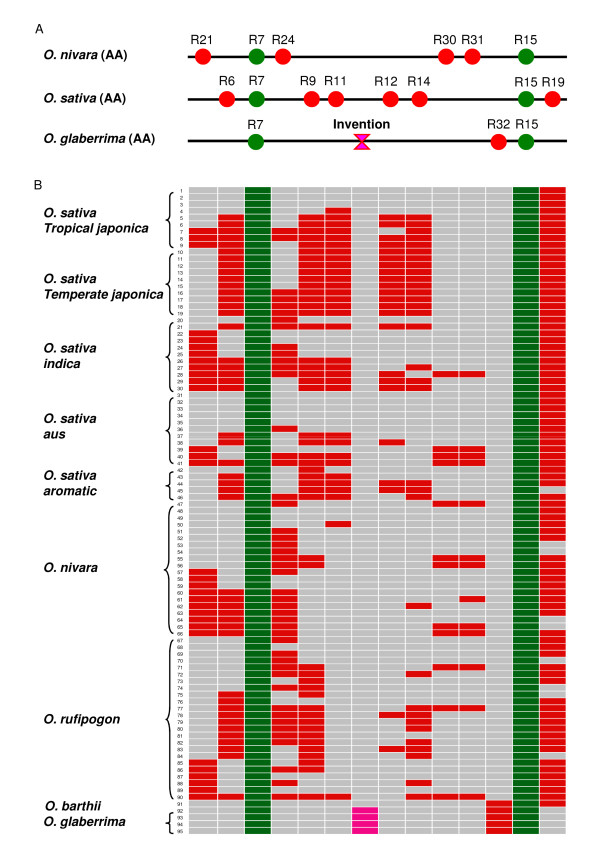
**Presence and absence of LTR-retrotransposons in the AA-genome variety populations**. (A) Elements investigated by PCR and their distribution in the three sequenced *Orp *regions. (B) The presence (red or green) or absence (grey) of individual LTR-retrotransposons detected by amplification of retrotransposon junctions. Varieties from top to bottom are listed in the same orders as shown in Additional file 2, Table S2.

Previous investigation of the *bz *genomic region in seven different maize inbred lines revealed remarkable variation in the maize genome, structure mediated by transposable elements [17,32]. Similar to the *bz *region, the *Orp *regions of the three *Oryza *AA genomes show a high level of polymorphisms of LTR-retrotransposon insertions (Figure 2). To further track whether a particular LTR-retrotransposon is present at high frequencies or fixed within a species/subspecies at population levels, we investigated the presence or absence of a set of LTR-retrotransposons identified in the *Orp *regions by PCR amplification of transposon insertion junctions in 95 AA genome varieties, following a protocol previously described by Devos et al. [1] (see details in Materials and Methods). These 95 varieties, including 46 *O. sativa*, 20 *O. nivara*, 24 *O. rufipogon *and 4 *O. glaberrima *and 1 *O. barthii *accessions (Additional file 2, Table S2) were chosen based on their geographic distribution and genetic diversity estimated by SSR and SNP markers [33-35]. The results of PCR analysis are illustrated in Figure 5 and Additional file 3, Figure S1. The primers designed for PCR analysis are listed in Additional file 4, Table S3.

R7 and R15, two representative LTR-retrotransposons shared by the three AA genomic regions, were detected in all the 94 AA genome varieties (Figure 5). In other words, these two insertions were fixed during the evolution and divergence of the AA genome species. It was estimated that these two elements were inserted approximately 2.1 and 1.2 million years ago. In general, LTR-retrotransposons tend to accumulate in low recombination heterochromatic regions where selection is expected to be less efficient in removing them [36]. Thus, the fixation of the insertion of these two elements inserted before the divergence of African and Asian rice lineages in the "originally" gene-rich *Orp *region with high rate of recombination [28] would suggest that these elements may have played or be playing an adaptive role.

We also investigated 11 LTR-retrotransposons that are not shared among the three sequenced *Orp *regions, including 6 (R6, R9, R11, R12, R14, and R19) unique to the *O. sativa *region, 4 (R21, R24, R30, and R31) unique to the *O. nivara *region, and 1 (R32) unique to the *O. glaberrima *region. These elements were relatively young (Additional file 1, Table S1) with an average age of 0.15 million years. R32 was found in the four *O. glaberrima *varieties, but absent in all Asian AA varieties, suggesting that this element was inserted into the African AA lineage after its divergence from the Asian AA lineage. R19 was found in 47 out of the 48 *O. sativa *varieties, being absent from a single *aromatic/GroupV *accession, and it was detected in 16 out of the 20 *O. nivara *varieties and 20 out of 24 *O. rufipogon *varieties. Interestingly, R6, R9, R11, R12 and R14, which distinguished Nipponbare from *O. nivara*, were fixed in the 10 *temperate japonica *varieties, while R21, R30 and R31, which were present in the sequenced *O. nivara *BAC, were completely absent from the *temperate japonica *subpopulation (Figure 5). A similar pattern was seen for R21, R30 and R31 (all absent), and for R6, R9 and R11 (all present) in the *aromatic/GroupV *accessions, which are known to be closely related to *temperate japonica *at the genetic level [37]. On the other hand, the *tropical japonica *varieties, as a group, were more similar to the *indica *and *aus *varieties across this region, with varying frequencies of LTRs matching those found in Nipponbare and/or *O. nivara*. Interestingly, the *indica *variety, 9311, which is known to have *japonica *parentage, shares a regional haplotye with *temperate japonica*, as do the two Indonesian *tropical japonica *varieties, Gotak Gatik and Trembese, while most of the others in this group carry the *O. nivara *alleles at R9, R112, R12 and R14. The *indica *subpopulation is almost fixed for the *O. nivara *allele at R21, and is highly variable across all other markers. The *aus *subpopulation is distinguished from the other *O. sativa *groups by a higher frequency of accessions carrying R30 and R31 and the complete absence of accessions carrying both R12 and R14. In these ways, *aus *more closely resembles *O. nivara *than other *O. sativa *varieties across this region. These observations are consistent with previous studies showing an intense genetic bottleneck in *temperate japonica *but greater variation in the *indica *and *aus *subgroups [33,38] and substantial admixture among the various subpopulations, most notably in *tropical japonica *[35].

Based on the presence/absence of all these LTR-retrotransposons, "phylogenetic relationships" of these varieties were analyzed. As shown in Additional file 5, Figure S2, the analysis clearly separated the African species, *O. glaberrima *and *O. barthii *(the proposed wild progenitor of *O. glaberrima*) from the Asian species, and the *indica *and *aus *subpopulations clustered at one end of the graph along with most of the *O. nivara *and some of the *O. rufipopgon *accessions, well separated from the majority of *aromatic/*Group V*, temperate *and *tropical japonica *varieties that clustered with a different set of *O. rufipogon *accessions. This analysis further identifies admixed *tropical japonica, indica *and *aus *varieties that cluster with several *O. nivara *and *O. rufipogon *accessions in the middle of the graph. Several *indica *varieties are seen clustering with the *japonica *group, reflecting the greater genetic variation and mixed parentage of many *indica *varieties, as previously noted for *c.v*. 9311. Thus, this graphical display reflects the taxonomy of these species and subspecies as established by SNP and/or SSR analyses, providing an interesting window on a highly variable region of the rice genome [33-35].

Several hypotheses can explain the observed lability of LTR-retrotransposons. 1) Since the regions are highly instable and plastic, there may be a high level of lineage sorting going on in present-day populations derived from a very diverse set of ancestral haplotypes. Each descendant population may inherit a large subset of the ancestral haplotypes, which continue to segregate in the descendants. In theory, over evolutionary time they should sort out such that each group has its own distinct haplotype/haplotypes that are more closely related to each other than to haplotypes from other species. However, because of the relatively short time that has elapsed for the speciation of the Asian AA genomes, the haplotypes of LTR-retrotransposons remain largely unsorted. 2) The lability can be explained by intra-specific and inter-specific introgression, which may have occurred during speciation of these genomes [35,39,40]. 3) Balancing selection for recent LTR-retrotransposon insertions may contribute to the high level of insertion polymorphisms, although adaptive selection and/or genetic bottlenecks affecting the two relatively old elements, R7 and R15, was suggested. Further investigation of a larger collection of wild and cultivated germplasm and more LTR-retrotransposon insertions at a larger genomic scale would help to reveal the dynamics of retention and/or removal of LTR-retrotransposons and their contributions to genomic diversity and speciation.

The inverted segment harboring genes 11.2, b1 and 12.2 in the sequenced *O. glaberrima *region (Figure 2) was detected in all other *O. glaberrima *accessions analyzed by PCR approach, but absent in the *O. barthii *accession and all Asian AA-genome *Oryza *species/subspecies (Figure 5 and Additional file 3, Figure S1). This suggests that the inversion occurred in African rice after its divergence from Asian rice. Because only a single *O. barthii *accession was included in this analysis, it remains unclear whether the inversion took place before or after the domestication of *O.glaberrima *from *O. barthii*.

The *Orp *region is located near the end of the short arm of rice chromosome 8, but harbors a high proportion of LTR-retrotransposons similar to that observed in the centromeric region of this same chromosome. Thus, it is likely that the region has recently switched from euchromatic to heterochromatic states.

## Conclusions

Our data indicate that the *Orp *genomic complex in rice cultivars and their wild progenitors have been recently, independently and concurrently formed from a gene-rich region by differential insertion of LTR-retrotransposons and genic rearrangement, and that the overall haplotype variation of LTR-retrotransposon insertions in this region echoes to the admixture pattern of genomic diversity and introgression of AA-genome populations/subpopulations revealed by genome-wide SSR and SNP genotyping, thus highlighting the evolutionary roles of LTR-retrotransposons in plant speciation and diversification. Genome-wide profiling of LTR-retrotransposon insertions among the AA-genome cultivars at larger population levels would enhance our understanding of the evolutionary processes and dynamics of the rice genomes.

## Methods

### Identification of BAC clones

The entire *Orp *region of *O. sativa *and it flanking 150-kb sequences from both ends of the region were searched against the BESs of other *Oryza *species generated by OMAP [19]. Single BAC clones meeting the following criteria: 1) at least one unique end; 2) both ends aligned to the extended *Orp *region of *O. sativa *in forward/reverse pairs; and 3) both ends spanning 100 to 500 kb of *O. sativa *sequences, were considered to be the orthologous segments from the respective *Oryza *species. As the major objective of this study was to target the genomic space corresponding to the hotspot of the transposable element accumulation and genic rearrangement in *O. sativa*, we only selected and analyzed a minimum number of clones from *Oryza *species that maximally cover the target region, as shown in Figure 1.

### BAC Sequencing

Shotgun libraries for selected BAC clones were constructed as described previously [41]. Subclones were sequenced from both directions using ABI PRISM BigDye Terminator Chemistry (Applied BioSystems, Foster, CA) and run on an ABI3730 capillary sequencer. BAC clones were sequenced at approximately 8-10-fold redundancies, and then were assembled and finished to standard high quality sequences (PHASE III) by primer walking [6]. The assemblies of sequenced BAC clones were confirmed by restriction map analysis similar to the method described by Dubcovsky et al. [41].

### Sequence annotation

Putative gene models were predicted using the FGENESH program with the monocot training set (http://www.softberry.com), and were further investigated to determine whether they are actually genes following described previously criteria [6]. Truncated gene fragments were identified by sequence homology comparison using BLAST2 [42], DOTTER [43] and CROSS_MATCH (http://www.phrap.org). LTR-retrotransposons were identified and classified as described previously [9]. DNA transposon fragments were identified by homology searches against the TIGR plant repeat database [44], GenBank non-redundant protein database, and *pack_MULE *database [45]. *Helitrons *were identified using a perl script described previously [46].

### Dating of segmental duplication and retrotransposon insertions

The alignments of homologous nucleotide sequences were generated by using ClustalX [47]. The dates of segmental duplication and amplification of LTR-retrotransposons were estimated as described previously [10]. The phylogenetic trees of duplicated genes were constructed based on pair-wise comparison of nucleotide sequences using the Kimura two-parameter method provided by MEGA4 program [48]. The Neighbor-Joining tree based on the presence/absence of LTR-retrotransposon insertions were obtained using MEGA4.

The genomic sequences generated in this study has been deposited in GeneBank (Nos. HM999006-HM999008)

## Authors' contributions

ZT, YaY, FL, YeY, and PJS carried out the BAC sequencing and haplotype variation experiments. ZT, PJS, RAW, SRM, JM and SAJ participated in data analysis, ZT, SRM and JM contributed to the manuscript preparation. All authors read and approved the final manuscript.

## Supplementary Material

Additional file 1 **Sequence annotation of different BACs in this study**. * 1605848 to 2060871 region of *O. sativa *subspecies *japonica *(c.v., Nipponbare) gemone sequece (Pseudomolecule 4.0). ** Intact-tsd, intact element flanked by target site duplication; solo-tsd; solo LTR flanked by target site duplication; intact-notsd, intact element without target site duplication; solo-notsd; solo LTR without target site duplication; &, internal sequence of LTR-retrotransposon; s, truncated fragment. Click here for file

Additional file 2 **The AA-genome Oryza varieties used in this study**. These 95 varieties, including 46 *O. sativa*, 20 *O. nivara*, 24 *O. rufipogon*, 4 *O. glaberrima*, and 1 *O. barthii *accessions, were chosen based on their geographic distribution and genetic diversity. Click here for file

Additional file 3 **PCR verification of a segmental inversion present in *O. glaberrima*, but absent in *O. sativa *and *O. nivara *varieties**. (A) Schematic primer design for amplification of inversion boundaries. (B) PCR products of primer pairs F1/R1 (panel b) and F2/R2 (panel c), and *Waxy *genes (panel a, control). Primers are shown in Additional file 4. Varieties are numbered according to their orders in Additional file 2. Click here for file

Additional file 4 **Primers used in this study**. Primers used to check the polymorphisms of LTR-retrotransposon insertions and the inversion in different species/subspecies. Click here for file

Additional file 5 **Neighbor-Joining "Phylogeny" of the AA-genome varieties constructed using MEGA4 based on the presence/absence of a set of LTR-retrotransposons in individual varieties as illustrated in Figure **5. Varieties are numbered according to their orders shown in Figure 5 and Additional file 2. Click here for file

## References

[B1] Devos KM Ma J Pontaroli AC Pratt LH Bennetzen JL Analysis and mapping of randomly chosen bacterial artificial chromosome clones from hexaploid bread wheat Proc Natl Acad Sci USA 2005 102 19243 19248 10.1073/pnas.0509473102 16357197 PMC1323192

[B2] Messing J Bennetzen JL Grass genome structure and evolution Genome Dyn 2008 4 41 56 full_text 18756076 10.1159/000126005

[B3] Lai J Ma J Swigonova Z Ramakrishna W Linton E Llaca V Tanyolac B Park YJ Jeong OY Bennetzen JL Gene loss and movement in the maize genome Genome Res 2004 14 1924 1931 10.1101/gr.2701104 15466290 PMC524416

[B4] Lai J Li Y Messing J Dooner HK Gene movement by Helitron transposons contributes to the haplotype variability of maize Proc Natl Acad Sci USA 2005 102 9068 9073 10.1073/pnas.0502923102 15951422 PMC1157042

[B5] Bruggmann R Bharti AK Gundlach H Lai J Young S Pontaroli AC Wei F Haberer G Fuks G Du C Uneven chromosome contraction and expansion in the maize genome Genome Res 2006 16 1241 1251 10.1101/gr.5338906 16902087 PMC1581433

[B6] Ma J SanMiguel P Lai J Messing J Bennetzen JL DNA rearrangement in orthologous *orp *regions of the maize, rice and sorghum genomes Genetics 2005 170 1209 1220 10.1534/genetics.105.040915 15834137 PMC1451190

[B7] Langham RJ Walsh J Dunn M Ko C Goff SA Freeling M Genomic duplication, fractionation and the origin of regulatory novelty Genetics 2004 166 935 945 10.1534/genetics.166.2.935 15020478 PMC1470742

[B8] Bennetzen JL Coleman C Liu R Ma J Ramakrishna W Consistent over-estimation of gene number in complex plant genomes Curr Opin Plant Biol 2004 7 732 736 10.1016/j.pbi.2004.09.003 15491923

[B9] Ma J Devos KM Bennetzen JL Analyses of LTR-retrotransposon structures reveal recent and rapid genomic DNA loss in rice Genome Res 2004 14 860 869 10.1101/gr.1466204 15078861 PMC479113

[B10] Ma J Bennetzen JL Rapid recent growth and divergence of rice nuclear genomes Proc Natl Acad Sci USA 2004 101 12404 12410 10.1073/pnas.0403715101 15240870 PMC515075

[B11] Han B Xue Y Genome-wide intraspecific DNA-sequence variations in rice Curr Opin Plant Biol 2003 6 134 138 10.1016/S1369-5266(03)00004-9 12667869

[B12] Fu H Dooner HK Intraspecific violation of genetic colinearity and its implications in maize Proc Natl Acad Sci USA 2002 99 9573 9578 12060715 10.1073/pnas.132259199 PMC123182

[B13] Dooner HK He L Maize genome structure variation: interplay between retrotransposon polymorphisms and genic recombination Plant Cell 2008 20 249 258 10.1105/tpc.107.057596 18296625 PMC2276454

[B14] Bennetzen JL Ma J Devos KM Mechanisms of recent genome size variation in flowering plants Ann Bot 2005 95 127 132 10.1093/aob/mci008 15596462 PMC4246713

[B15] Vitte C Bennetzen JL Analysis of retrotransposon structural diversity uncovers properties and propensities in angiosperm genome evolution Proc Natl Acad Sci USA 2006 103 17638 17643 10.1073/pnas.0605618103 17101966 PMC1693799

[B16] Ilic K SanMiguel PJ Bennetzen JL A complex history of rearrangement in an orthologous region of the maize, sorghum, and rice genomes Proc Natl Acad Sci USA 2003 100 12265 12270 10.1073/pnas.1434476100 14530400 PMC218747

[B17] Wang Q Dooner HK Remarkable variation in maize genome structure inferred from haplotype diversity at the *bz *locus Proc Natl Acad Sci USA 2006 103 17644 17649 10.1073/pnas.0603080103 17101975 PMC1693800

[B18] International Rice Genome Sequencing Project The map-based sequence of the rice genome Nature 2005 436 793 800 10.1038/nature03895 16100779

[B19] Wing RA Ammiraju JS Luo M Kim H Yu Y Kudrna D Goicoechea JL Wang W Nelson W Rao K The *Oryza *map alignment project: the golden path to unlocking the genetic potential of wild rice species Plant Mol Biol 2005 59 53 62 10.1007/s11103-004-6237-x 16217601

[B20] Ammiraju JS Lu F Sanyal A Yu Y Song X Jiang N Pontaroli AC Rambo T Currie J Collura K Dynamic evolution of *Oryza *genomes is revealed by comparative genomic analysis of a genus-wide vertical data set Plant Cell 2008 20 3191 3209 10.1105/tpc.108.063727 19098269 PMC2630430

[B21] Lu F Ammiraju JS Sanyal A Zhang S Song R Chen J Li G Sui Y Song X Cheng Z Comparative sequence analysis of *MONOCULM1-*orthologous regions in 14 *Oryza *genomes Proc Natl Acad Sci USA 2009 106 2071 2076 10.1073/pnas.0812798106 19164767 PMC2629783

[B22] Sanyal A Jetty AS Lu F Yu Y Rambo T Currie J Kollura K Kim HR Chen J Ma J Orthologous comparisons of the *Hd1 *region across genera reveal Hd1 gene lability within diploid *Oryza *species and disruptions to microsynteny in sorghum Mol Biol Evol 2010 27 2487 2506 10.1093/molbev/msq133 20522726

[B23] Ge S Sang T Lu BR Hong DY Phylogeny of rice genomes with emphasis on origins of allotetraploid species Proc Natl Acad Sci USA 1999 96 14400 14405 10.1073/pnas.96.25.14400 10588717 PMC24448

[B24] Ma J Jackson SA Retrotransposon accumulation and satellite amplification mediated by segmental duplication facilitate centromere expansion in rice Genome Res 2006 16 251 259 10.1101/gr.4583106 16354755 PMC1361721

[B25] Roulin A Chaparro C Piegu B Jackson S Panaud O Paleogenomic analysis of the short arm of chromosome 3 reveals the history of the African and Asian progenitors of cultivated rices Genome Biol Evol 2010 2 132 139 10.1093/gbe/evq005 20333229 PMC2839358

[B26] Devos KM Brown JK Bennetzen JL Genome size reduction through illegitimate recombination counteracts genome expansion in *Arabidopsis* Genome Res 2002 12 1075 1079 10.1101/gr.132102 12097344 PMC186626

[B27] McCarthy EM Liu J Lizhi G McDonald JF Long terminal repeat retrotransposons of *Oryza sativa* Genome Biol 2002 3 RESEARCH0053 10.1186/gb-2002-3-10-research0053 12372141 PMC134482

[B28] Tian Z Rizzon C Du J Zhu L Bennetzen JL Jackson SA Gaut BS Ma J Do genetic recombination and gene density shape the pattern of DNA elimination in rice long terminal repeat retrotransposons? Genome Res 2009 19 2221 2230 10.1101/gr.083899.108 19789376 PMC2792168

[B29] Alexander RP Fang G Rozowsky J Snyder M Gerstein MB Annotating non-coding regions of the genome Nat Rev Genet 2010 11 559 571 10.1038/nrg2814 20628352

[B30] Kashkush K Feldman M Levy AA Gene loss, silencing and activation in a newly synthesized wheat allotetraploid Genetics 2002 160 1651 1659 11973318 10.1093/genetics/160.4.1651 PMC1462064

[B31] Ginzburg LR Bingham PM Yoo S On the theory of speciation induced by transposable elements Genetics 1984 107 331 341 6329903 10.1093/genetics/107.2.331 PMC1202326

[B32] He L Dooner HK Haplotype structure strongly affects recombination in a maize genetic interval polymorphic for Helitron and retrotransposon insertions Proc Natl Acad Sci USA 2009 106 8410 8416 10.1073/pnas.0902972106 19416860 PMC2688972

[B33] Garris AJ Tai TH Coburn J Kresovich S McCouch S Genetic structure and diversity in *Oryza sativa *L Genetics 2005 169 1631 1638 10.1534/genetics.104.035642 15654106 PMC1449546

[B34] Semon M Nielsen R Jones MP McCouch SR The population structure of African cultivated rice *Oryza glaberrima *(Steud.): evidence for elevated levels of linkage disequilibrium caused by admixture with *O. sativa *and ecological adaptation Genetics 2005 169 1639 1647 10.1534/genetics.104.033175 15545652 PMC1449534

[B35] Zhao K Wright M Kimball J Eizenga G McClung A Kovach M Tyagi W Ali ML Tung CW Reynolds A Genomic diversity and introgression in *O. sativa *reveal the impact of domestication and breeding on the rice genome PLoS One 2010 5 e10780 10.1371/journal.pone.0010780 20520727 PMC2875394

[B36] Gonzalez J Macpherson JM Petrov DA A recent adaptive transposable element insertion near highly conserved developmental loci in *Drosophila melanogaster* Mol Biol Evol 2009 26 1949 1961 10.1093/molbev/msp107 19458110 PMC2734154

[B37] Kovach MJ Calingacion MN Fitzgerald MA McCouch SR The origin and evolution of fragrance in rice (*Oryza sativa *L.) Proc Natl Acad Sci USA 2009 106 14444 14449 10.1073/pnas.0904077106 19706531 PMC2732888

[B38] Caicedo AL Williamson SH Hernandez RD Boyko A Fledel-Alon A York TL Polato NR Olsen KM Nielsen R McCouch SR Genome-wide patterns of nucleotide polymorphism in domesticated rice PLoS Genet 2007 3 1745 1756 10.1371/journal.pgen.0030163 17907810 PMC1994709

[B39] Kovach MJ McCouch SR Leveraging natural diversity: back through the bottleneck Curr Opin Plant Biol 2008 11 193 200 10.1016/j.pbi.2007.12.006 18313975

[B40] Vaughan DA Lu B-R Tomooka N The evolving story of rice evolution Plant Science 2008 174 394 408

[B41] Dubcovsky J Ramakrishna W SanMiguel PJ Busso CS Yan L Shiloff BA Bennetzen JL Comparative sequence analysis of colinear barley and rice bacterial artificial chromosomes Plant Physiol 2001 125 1342 1353 10.1104/pp.125.3.1342 11244114 PMC65613

[B42] Tatusova TA Madden TL BLAST 2 Sequences, a new tool for comparing protein and nucleotide sequences FEMS Microbiol Lett 1999 174 247 250 10.1111/j.1574-6968.1999.tb13575.x 10339815

[B43] Sonnhammer EL Durbin R A dot-matrix program with dynamic threshold control suited for genomic DNA and protein sequence analysis Gene 1995 167 GC1 10 10.1016/0378-1119(95)00714-8 8566757

[B44] Ouyang S Zhu W Hamilton J Lin H Campbell M Childs K Thibaud-Nissen F Malek RL Lee Y Zheng L The TIGR Rice Genome Annotation Resource: improvements and new features Nucleic Acids Res 2007 35 D883 887 10.1093/nar/gkl976 17145706 PMC1751532

[B45] Jiang N Bao Z Zhang X Eddy SR Wessler SR Pack-MULE transposable elements mediate gene evolution in plants Nature 2004 431 569 573 10.1038/nature02953 15457261

[B46] Yang L Bennetzen JL Distribution, diversity, evolution, and survival of Helitrons in the maize genome Proc Natl Acad Sci USA 2009 106 19922 19927 19926865 10.1073/pnas.0908008106 PMC2785268

[B47] Thompson JD Gibson TJ Plewniak F Jeanmougin F Higgins DG The CLUSTAL_X windows interface: flexible strategies for multiple sequence alignment aided by quality analysis tools Nucleic Acids Res 1997 25 4876 4882 10.1093/nar/25.24.4876 9396791 PMC147148

[B48] Tamura K Dudley J Nei M Kumar S MEGA4: Molecular Evolutionary Genetics Analysis (MEGA) software version 4.0 Mol Biol Evol 2007 24 1596 1599 10.1093/molbev/msm092 17488738

