# Supplementary material for: Exceptional lability of a genomic complex in rice and its close relatives revealed by interspecific and intraspecific comparison and population analysis
Source: BMC Genomics. 2011 Mar 8;12:142. doi: 10.1186/1471-2164-12-142 (PMC3060143; doi:10.1186/1471-2164-12-142)
Supplement: Additional file 1 — Sequence annotation of different BACs in this study. * 1605848 to 2060871 region of O. sativa subspecies japonica (c.v., Nipponbare) gemone sequece (Pseudomolecule 4.0). ** Intact-tsd, intact element flanked by target site duplication; solo-tsd; solo LTR flanked by target site duplication; intact-notsd, intact element without target site duplication; solo-notsd; solo LTR without target site duplication; &, internal sequence of LTR-retrotransposon; s, truncated fragment. [file 1471-2164-12-142-S1.DOC]

| **Table S1. Sequence annotation of different BACs in this study** | | | | | | |
| --- | --- | --- | --- | --- | --- | --- |
|
| Species | BAC or contig | Annotation | From | To | Age (MYs) | Note^**^ |
| *O. sativa* (AA) | orp_chr8_v4* | H-0 | 87017 | 87521 |  | *Helitron* |
| *O. sativa* (AA) | orp_chr8_v4 | gene 21 | 117196 | 122312 |  |  |
| *O. sativa* (AA) | orp_chr8_v4 | T-1 | 126711 | 135804 |  |  |
| *O. sativa* (AA) | orp_chr8_v4 | gene 17 | 137821 | 141779 |  |  |
| *O. sativa* (AA) | orp_chr8_v4 | gene 18 | 143000 | 145090 |  |  |
| *O. sativa* (AA) | orp_chr8_v4 | gene 19 | 146282 | 148491 |  |  |
| *O. sativa* (AA) | orp_chr8_v4 | gene 20 | 149148 | 150057 |  |  |
| *O. sativa* (AA) | orp_chr8_v4 | gene 14 | 150867 | 152354 |  |  |
| *O. sativa* (AA) | orp_chr8_v4 | R-1 | 156351 | 157051 |  | solo-tsd |
| *O. sativa* (AA) | orp_chr8_v4 | T-2 | 160055 | 170814 |  |  |
| *O. sativa* (AA) | orp_chr8_v4 | Mule | 171955 | 180634 |  |  |
| *O. sativa* (AA) | orp_chr8_v4 | R-2 | 189780 | 192768 | 3.408 | intact-tsd |
| *O. sativa* (AA) | orp_chr8_v4 | T-3 | 195293 | 195714 |  |  |
| *O. sativa* (AA) | orp_chr8_v4 | T-4 | 196503 | 197753 |  |  |
| *O. sativa* (AA) | orp_chr8_v4 | R-f | 197635 | 199581 |  |  |
| *O. sativa* (AA) | orp_chr8_v4 | R-3 | 198030 | 199524 |  | solo-tsd |
| *O. sativa* (AA) | orp_chr8_v4 | H-1 | 205398 | 208315 |  | *Helitron* |
| *O. sativa* (AA) | orp_chr8_v4 | R-4 | 209901 | 216306 | 0.950 | intact-tsd |
| *O. sativa* (AA) | orp_chr8_v4 | R-5 | 216976 | 217680 |  | solo-tsd |
| *O. sativa* (AA) | orp_chr8_v4 | T-5 | 225209 | 225447 |  |  |
| *O. sativa* (AA) | orp_chr8_v4 | gene a.2 | 237200 | 239001 |  |  |
| *O. sativa* (AA) | orp_chr8_v4 | R-6 | 241244 | 246231 | 0.135 | intact-tsd |
| *O. sativa* (AA) | orp_chr8_v4 | gene 12.3 | 246352 | 248456 |  |  |
| *O. sativa* (AA) | orp_chr8_v4 | gene b.2 | 248550 | 249779 |  |  |
| *O. sativa* (AA) | orp_chr8_v4 | MITE | 250430 | 251168 |  |  |
| *O. sativa* (AA) | orp_chr8_v4 | R-7 | 255826 | 274281 | 2.111 | intact-tsd |
| *O. sativa* (AA) | orp_chr8_v4 | R-8 | 260254 | 271664 | 0.862 | intact-tsd |
| *O. sativa* (AA) | orp_chr8_v4 | R-9 | 274859 | 290723 | 0.281 | intact-tsd |
| *O. sativa* (AA) | orp_chr8_v4 | R-10 | 277030 | 280190 |  | solo-tsd |
| *O. sativa* (AA) | orp_chr8_v4 | R-11 | 293273 | 304837 | 0.088 | intact-tsd |
| *O. sativa* (AA) | orp_chr8_v4 | gene a.1 | 313655 | 316433 |  |  |
| *O. sativa* (AA) | orp_chr8_v4 | gene 12.2-P1 | 320114 | 321187 |  |  |
| *O. sativa* (AA) | orp_chr8_v4 | gene 12.2-P2 | 329220 | 330079 |  |  |
| *O. sativa* (AA) | orp_chr8_v4 | R-12 | 321188 | 329220 | 0.092 | intact-tsd |
| *O. sativa* (AA) | orp_chr8_v4 | gene b.1 | 330173 | 330717 |  |  |
| *O. sativa* (AA) | orp_chr8_v4 | gene 11.2 | 330775 | 334472 |  |  |
| *O. sativa* (AA) | orp_chr8_v4 | R-f-13 | 334695 | 336409 |  |  |
| *O. sativa* (AA) | orp_chr8_v4 | R-14 | 337575 | 343982 | 0.365 | intact-tsd |
| *O. sativa* (AA) | orp_chr8_v4 | mt | 345595 | 346180 |  |  |
| *O. sativa* (AA) | orp_chr8_v4 | T-f-6 | 346423 | 347654 |  |  |
| *O. sativa* (AA) | orp_chr8_v4 | H-2 | 350172 | 350942 |  | *Helitron* |
| *O. sativa* (AA) | orp_chr8_v4 | R-15 | 351286 | 369666 | 1.204 | intact-tsd |
| *O. sativa* (AA) | orp_chr8_v4 | R-16 | 353744 | 357065 |  | solo-tsd |
| *O. sativa* (AA) | orp_chr8_v4 | R-17 | 358449 | 361781 |  | solo-tsd |
| *O. sativa* (AA) | orp_chr8_v4 | H3-P1 | 371397 | 371697 |  | *Helitron* |
| *O. sativa* (AA) | orp_chr8_v4 | R-18 | 371698 | 376148 | 0.835 | intact-tsd |
| *O. sativa* (AA) | orp_chr8_v4 | H3-P2 | 376149 | 376451 |  | *Helitron* |
| *O. sativa* (AA) | orp_chr8_v4 | R-19 | 376651 | 379768 |  | solo-tsd |
| *O. sativa* (AA) | orp_chr8_v4 | gene 12.1 | 388188 | 391983 |  |  |
| *O. sativa* (AA) | orp_chr8_v4 | gene 11.1 | 393333 | 398383 |  |  |
| *O. sativa* (AA) | orp_chr8_v4 | gene 10 | 401299 | 404532 |  |  |
| *O. sativa* (AA) | orp_chr8_v4 | gene 9 | 405267 | 406061 |  |  |
| *O. sativa* (AA) | orp_chr8_v4 | gene 8 | 407101 | 408533 |  |  |
| *O. sativa* (AA) | orp_chr8_v4 | gene 5 | 416048 | 419317 |  |  |
| *O. sativa* (AA) | orp_chr8_v4 | gene 3 | 422942 | 425977 |  |  |
| *O. sativa* (AA) | orp_chr8_v4 | gene 2 | 428201 | 430660 |  |  |
| *O. sativa* (AA) | orp_chr8_v4 | T | 434107 | 434846 |  |  |
| *O. sativa* (AA) | orp_chr8_v4 | T | 441268 | 442514 |  |  |
| *O. sativa* (AA) | orp_chr8_v4 | R | 442515 | 446420 |  | solo-tsd |
| *O. sativa* (AA) | orp_chr8_v4 | T | 446421 | 447049 |  |  |
| *O. nivara* (AA) | OR_BBa0014L06 | T-3 | 9517 | 9925 |  |  |
| *O. nivara* (AA) | OR_BBa0014L06 | R-3 | 12240 | 13734 |  | solo-tsd |
| *O. nivara* (AA) | OR_BBa0014L06 | H-1 | 19636 | 22552 |  | *Helitron* |
| *O. nivara* (AA) | OR_BBa0014L06 | R-4 | 24142 | 37341 | 1.165 | intact-tsd |
| *O. nivara* (AA) | OR_BBa0014L06 | R-20 | 24805 | 28204 |  | solo-tsd |
| *O. nivara* (AA) | OR_BBa0014L06 | R-21 | 30020 | 33186 |  | solo-tsd |
| *O. nivara* (AA) | OR_BBa0014L06 | R-5 | 38011 | 38715 |  | solo-tsd |
| *O. nivara* (AA) | OR_BBa0014L06 | R-22 | 38918 | 51782 | 0.250 | intact-tsd |
| *O. nivara* (AA) | OR_BBa0014L06 | R-23 | 54811 | 61251 | 0.000 | intact-notsd |
| *O. nivara* (AA) | OR_BBa0014L06 | T-5 | 65849 | 66087 |  |  |
| *O. nivara* (AA) | OR_BBa0014L06 | gene a.2 | 75951 | 77744 |  |  |
| *O. nivara* (AA) | OR_BBa0014L06 | gene 12.3 | 80809 | 82912 |  |  |
| *O. nivara* (AA) | OR_BBa0014L06 | gene b.2 | 83006 | 84624 |  |  |
| *O. nivara* (AA) | OR_BBa0014L06 | R-7 | 89465 | 147608 | 1.811 | ltr1&ltr2-s |
| *O. nivara* (AA) | OR_BBa0014L06 | R-8 | 93917 | 145339 | 1.085 | intact-tsd |
| *O. nivara* (AA) | OR_BBa0014L06 | R-24 | 102526 | 105612 |  | solo-tsd |
| *O. nivara* (AA) | OR_BBa0014L06 | R-25 | 107180 | 110581 |  | solo-tsd |
| *O. nivara* (AA) | OR_BBa0014L06 | R-26 | 111578 | 145065 |  | solo-tsd |
| *O. nivara* (AA) | OR_BBa0014L06 | R-27 | 112837 | 143333 | 0.200 | intact |
| *O. nivara* (AA) | OR_BBa0014L06 | R-28 | 118629 | 130305 | 0.012 | ltr1&ltr2-s |
| *O. nivara* (AA) | OR_BBa0014L06 | R-29 | 133817 | 142149 | 0.081 | intact-tsd |
| *O. nivara* (AA) | OR_BBa0014L06 | gene a.1 | 155402 | 158177 |  |  |
| *O. nivara* (AA) | OR_BBa0014L06 | gene 12.2 | 161917 | 163791 |  |  |
| *O. nivara* (AA) | OR_BBa0014L06 | gene b.1 | 163885 | 164426 |  |  |
| *O. nivara* (AA) | OR_BBa0014L06 | gene 11.2-P1 | 164487 | 165202 |  |  |
| *O. nivara* (AA) | OR_BBa0014L06 | R-30 | 165203 | 170440 | 0.277 | intact-tsd |
| *O. nivara* (AA) | OR_BBa0014L06 | gene 11.2-P2 | 170441 | 173427 |  |  |
| *O. nivara* (AA) | OR_BBa0014L06 | R-31 | 175582 | 178871 |  | solo-tsd |
| *O. nivara* (AA) | OR_BBa0014L06 | H-2 | 184873 | 185606 |  | *Helitron* |
| *O. nivara* (AA) | OR_BBa0014L06 | R-15 | 185950 | 200905 | 1.338 | intact-tsd |
| *O. nivara* (AA) | OR_BBa0014L06 | R-16 | 188408 | 191690 |  | solo-tsd |
| *O. nivara* (AA) | OR_BBa0014L06 | H-3 | 202670 | 207723 |  | *Helitron* |
| *O. nivara* (AA) | OR_BBa0014L06 | R-18 | 202972 | 207420 | 1.085 | intact-tsd |
| *O. glabberima* (AA) | OGBBa0001L21 | gene 21-p | 1 | 297 |  |  |
| *O. glabberima* (AA) | OGBBa0001L21 | gene 17 | 6591 | 10558 |  |  |
| *O. glabberima* (AA) | OGBBa0001L21 | gene 18 | 11778 | 13865 |  |  |
| *O. glabberima* (AA) | OGBBa0001L21 | gene 19 | 15057 | 17265 |  |  |
| *O. glabberima* (AA) | OGBBa0001L21 | gene 20 | 17922 | 18829 |  |  |
| *O. glabberima* (AA) | OGBBa0001L21 | gene 14 | 19644 | 21131 |  |  |
| *O. glabberima* (AA) | OGBBa0001L21 | R-1 | 25162 | 25867 |  | solo-tsd |
| *O. glabberima* (AA) | OGBBa0001L21 | R-2 | 38391 | 38863 |  | solo-tsd |
| *O. glabberima* (AA) | OGBBa0001L21 | gene a.2 | 44304 | 46098 |  |  |
| *O. glabberima* (AA) | OGBBa0001L21 | R-7 | 47578 | 63600 |  | &ltr2 |
| *O. glabberima* (AA) | OGBBa0001L21 | R-8 | 50873 | 62273 |  | ltr1&ltr2-s |
| *O. glabberima* (AA) | OGBBa0001L21 | R-15.1 | 73285 | 76260 |  | &ltr2 |
| *O. glabberima* (AA) | OGBBa0001L21 | R-16.1 | 73285 | 73774 |  | s-ltr |
| *O. glabberima* (AA) | OGBBa0001L21 | H-2 | 76604 | 77108 |  | *Helitron* |
| *O. glabberima* (AA) | OGBBa0001L21 | gene 11 | 84360 | 87740 |  | INVERED |
| *O. glabberima* (AA) | OGBBa0001L21 | gene b | 87801 | 88342 |  | INVERED |
| *O. glabberima* (AA) | OGBBa0001L21 | gene 12 | 88436 | 90312 |  | INVERED |
| *O. glabberima* (AA) | OGBBa0001L21 | gene a -p | 94046 | 94684 |  | INVERED |
| *O. glabberima* (AA) | OGBBa0001L21 | R-16.2 | 94910 | 103009 |  | &ltr2 |
| *O. glabberima* (AA) | OGBBa0001L21 | R-15.2 | 103010 | 116067 |  | &ltr2 |
| *O. glabberima* (AA) | OGBBa0001L21 | R-32 | 111956 | 115728 |  | solo-tsd |
| *O. glabberima* (AA) | OGBBa0001L21 | H-3 | 117892 | 118193 |  | *Helitron* |
| *O. glabberima* (AA) | OGBBa0001L21 | R-18 | 118194 | 118998 |  | solo-tsd |
| *O. glabberima* (AA) | OGBBa0001L21 | gene 12 | 128229 | 132024 |  |  |
| *O. glabberima* (AA) | OGBBa0001L21 | gene 11 | 133907 | 138954 |  |  |
| *O. glabberima* (AA) | OGBBa0001L21 | gene 10 | 141888 | 144917 |  |  |
| *O. glabberima* (AA) | OGBBa0001L21 | gene 9 | 145664 | 146449 |  |  |
| *O. glabberima* (AA) | OGBBa0001L21 | gene 8 | 147515 | 148842 |  |  |
| *O. glabberima* (AA) | OGBBa0001L21 | gene 5 | 154047 | 157306 |  |  |
| *O. glabberima* (AA) | OGBBa0001L21 | gene 3 | 160429 | 163377 |  |  |
| *O. glabberima* (AA) | OGBBa0001L21 | gene 2 | 165735 | 168197 |  |  |
| *O. glabberima* (AA) | OGBBa0001L21 | T | 171692 | 172431 |  |  |
| *O. glabberima* (AA) | OGBBa0001L21 | T | 179976 | 181845 |  |  |
| *O. punctata* (BB) | OPBa0008J05rc | gene 21 | 38294 | 42334 |  |  |
| *O. punctata* (BB) | OPBa0008J05rc | gene 17 | 50739 | 54534 |  |  |
| *O. punctata* (BB) | OPBa0008J05rc | gene 18 | 55553 | 57576 |  |  |
| *O. punctata* (BB) | OPBa0008J05rc | gene 19 | 58980 | 61199 |  |  |
| *O. punctata* (BB) | OPBa0008J05rc | gene 20 | 61935 | 62606 |  |  |
| *O. punctata* (BB) | OPBa0008J05rc | gene 14 | 63485 | 64978 |  |  |
| *O. punctata* (BB) | OPBa0008J05rc | R-33 | 87111 | 100768 | 0.042 | intact-tsd |
| *O. punctata* (BB) | OPBa0008J05rc | gene a.2 | 107530 | 109295 |  |  |
| *O. punctata* (BB) | OPBa0008J05rc | gene 12.2.4 | 110672 | 110831 |  |  |
| *O. punctata* (BB) | OPBa0008J05rc | gene 11.2.4 | 112094 | 115963 |  |  |
| *O. punctata* (BB) | OPBa0008J05rc | gene 12.2.3 | 122206 | 122365 |  |  |
| *O. punctata* (BB) | OPBa0008J05rc | gene 11.2.3 | 123628 | 127497 |  |  |
| *O. punctata* (BB) | OPBa0008J05rc | gene 11.2.2 | 133056 | 138925 |  |  |
| *O. punctata* (BB) | OPBa0008J05rc | gene 12.2.2 | 133634 | 133793 |  |  |
| *O. punctata* (BB) | OPBa0008J05rc | gene 12.2.1 | 145168 | 145327 |  |  |
| *O. punctata* (BB) | OPBa0008J05rc | gene 11.2.1 | 146590 | 148034 |  |  |
| *O. punctata* (BB) | OPBa0008J05rc | gene 12.1 | 160084 | 163793 |  |  |
| *O. punctata* (BB) | OPBa0008J05rc | gene 11.1 | 165325 | 170843 |  |  |
| *O. punctata* (BB) | OPBa0008J05rc | gene 10 | 174992 | 178270 |  |  |
| *O. punctata* (BB) | OPBa0008J05rc | gene 9 | 178976 | 179683 |  |  |
| *O. punctata* (BB) | OPBa0008J05rc | gene 8 | 180729 | 182166 |  |  |
| *O. punctata* (BB) | OPBa0008J05rc | gene 5-p | 189651 | 192133 |  |  |
| *O. punctata* (BB) | OPBa0008J05rc | T-7 | 190440 | 191306 |  |  |
| * 1605848 to 2060871 region of *O. sativa* subspecies *japonica* (c.v., Nipponbare) genome sequence (Pseudomolecule 4.0). | | | | | | |
| ** Intact-tsd, intact element flanked by target site duplication; solo-tsd; solo LTR flanked by target site duplication; | | | | | | |
| intact-notsd, intact element without target site duplication; solo-notsd, solo LTR without target site duplication; &, | | | | | | |
| internal sequence of LTR-retrotransposon; s, truncated fragment. | | | | | | |
