# Supplementary material for: Exceptional lability of a genomic complex in rice and its close relatives revealed by interspecific and intraspecific comparison and population analysis
Source: BMC Genomics. 2011 Mar 8;12:142. doi: 10.1186/1471-2164-12-142 (PMC3060143; doi:10.1186/1471-2164-12-142)
Supplement: Additional file 2 — The AA-genome Oryza varieties used in this study. These 95 varieties, including 46 O. sativa, 20 O. nivara, 24 O. rufipogon, 4 O. glaberrima, and 1 O. barthii accessions, were chosen based on their geographic distribution and genetic diversity. [file 1471-2164-12-142-S2.DOC]

| **Table S2. The AA-genome *Oryza* varieties used in this study** | | | | | |
| --- | --- | --- | --- | --- | --- |
| Name | IRGC # | Cornell Rice # | Species | Origin | # n Fig S2 |
| Arias | IRGC 43325 | RA_6236 | *O. sativa Tropical japonica* | Indonesia | 1 |
| Asse Y Pung | IRGC 6949 | RA_6335 | *O. sativa Tropical japonica* | Philippines | 2 |
| Miriti | IRGC 25901 | RA_6248 | *O. sativa Tropical japonica* | Bangladesh | 3 |
| Azucena | IRGC 328 | RA_7737 | *O. sativa Tropical japonica* | Philippines | 4 |
| Gotak Gatik | IRGC 43397 | RA_6241 | *O. sativa Tropical japonica* | Indonesia | 5 |
| Trembese | IRGC 43675 | RA_6262 | *O. sativa Tropical japonica* | Indonesia | 6 |
| Moroberekan | IRGC 12048 | RA_7740 | *O. sativa Tropical japonica* | Guinea | 7 |
| Kotobuki Mochi | IRGC 2545 | RA_6203 | *O. sativa Tropical japonica* | Japan | 8 |
| Lemont | IRGC 66756 | RA_6270 | *O. sativa Tropical japonica* | USA | 9 |
| Mansaku | IRGC 8191 | RA_6284 | *O. sativa Temperate japonica* | Japan | 10 |
| Chinese |  | RA_6321 | *O. sativa Temperate japonica* | China | 11 |
| Agostana | IRGC 3135 | RA_6349 | *O. sativa Temperate japonica* | Italy | 12 |
| Geumbyeo |  | RA_6361 | *O. sativa Temperate japonica* | Korea | 13 |
| Koshihikari |  | RA_6336 | *O. sativa Temperate japonica* | Japan | 14 |
| Nipponbare | IRGC 12731 | RA_7741 | *O. sativa Temperate japonica* | Japan | 15 |
| Norin 20 | IRGC 418 | RA_6323 | *O. sativa Temperate japonica* | Japan | 16 |
| Shinriki |  | RA_6334 | *O. sativa Temperate japonica* | Japan | 17 |
| Shoemed | IRGC 5757 | RA_6310 | *O. sativa Temperate japonica* | USA | 18 |
| Suweon 362 |  | RA_6337 | *O. sativa Temperate japonica* | Korea | 19 |
| Mudgo | IRGC 6663 | RA_6316 | *O. sativa indica* | India | 20 |
| 9311 |  | RA_6193 | *O. sativa indica* | China | 21 |
| Teqing |  | RA_6314 | *O. sativa indica* | China | 22 |
| Chiemchanh | IRGC 10214 | RA_6221 | *O. sativa indica* | Vietnam | 23 |
| Guan-Yin-Tsan | IRGC 51300 | RA_6247 | *O. sativa indica* | China | 24 |
| Jaya | IRGC11099 | RA_6319 | *O. sativa indica* | India | 25 |
| Dee Geo Woo Ge |  | RA_6340 | *O. sativa indica* | Taiwan | 26 |
| Chau | IRGC 56036 | RA_6252 | *O. sativa indica* | Vietnam | 27 |
| Rathawee | IRGC 8952 | RA_6218 | *O. sativa indica* | Srilanka | 28 |
| Taducan |  | RA_6357 | *O. sativa indica* | India | 29 |
| IR 64 |  | RA_7746 | *O. sativa indica* | Philippines | 30 |
| Basmati 1 | IRGC 27798 | RA_6226 | *O. sativa Aus* | Pakistan | 31 |
| Champa Tong 54 | IRGC 30238 | RA_6229 | *O. sativa Aus* | Thailand | 32 |
| DV85 | IRGC 8839 | RA_6332 | *O. sativa Aus* | Bangladesh | 33 |
| Kasalath | HO 1195 | RA_6338 | *O. sativa Aus* | Japan | 34 |
| ARC 10352 | IRGC 12440 | RA_6258 | *O. sativa Aus* | India | 35 |
| BJ 1 | IRGC 45195 | RA_6341 | *O. sativa Aus* | India | 36 |
| Khao Gaew | IRGC 24224 | RA_6297 | *O. sativa Aus* | Thailand | 37 |
| BLACK GORA | IRGC 40275 | RA_6274 | *O. sativa Aus* | India | 38 |
| Dhala Shaitta | IRGC 3686 | RA_6350 | *O. sativa Aus* | Bangladesh | 39 |
| T1 | IRGC 6294 | RA_6205 | *O. sativa Aus* | India | 40 |
| Phudugey | IRGC 32399 | RA_6232 | *O. sativa Aus* | Bhutan | 41 |
| Basmati | IRGC 27805 | RA_6333 | *O. sativa aromatic* | Pakistan | 42 |
| Basmati 217 | IRGC 53637 | RA_6266 | *O. sativa aromatic* | India | 43 |
| Bico Branco | IRGC 38994 | RA_6235 | *O. sativa aromatic* | Brazil | 44 |
| Kitrana 508 | IRGC 12793 | RA_6275 | *O. sativa aromatic* | Madagaskar | 45 |
| Dom-Sofid | IRGC 12880 | RA_7742 | *O. sativa aromatic* | Iran | 46 |
|  | IRGC 80759 | RA_6976 | *O. nivara* | Myanmar | 47 |
|  | IRGC 105428 | RA_7056 | *O. nivara* | SriLanka | 48 |
|  | IRGC 106155 | RA_7092 | *O. nivara* | Laos | 49 |
|  | IRGC 105879 | RA_7075 | *O. nivara* | Bangladesh | 50 |
|  | IRGC103835 | RA_7899 | *O. nivara* | Bangladesh | 51 |
|  | IRGC 103836 | RA_7900 | *O. nivara* | Bangladesh | 52 |
|  | IRGC 104650 | RA_7909 | *O. nivara* | Thailand | 53 |
|  | IRGC 104687 | RA_7911 | *O. nivara* | India | 54 |
|  | IRGC 105740 | RA_7938 | *O. nivara* | Cambodia | 55 |
|  | IRGC 106396 | RA_7954 | *O. nivara* | Mayanmar | 56 |
|  | IRGC 103821 | RA_7023 | *O. nivara* | China | 57 |
|  | IRGC 103824 | RA_7025 | *O. nivara* | China | 58 |
|  | IRGC 104644 | RA_7042 | *O. nivara* | Thailand | 59 |
|  | IRGC 93183 | RA_7012 | *O. nivara* | Nepal | 60 |
|  | IRGC 100195 | RA_7018 | *O. nivara* | Myanmar | 61 |
|  | IRGC 103813 | RA_7893 | *O. nivara* | China | 62 |
|  | IRGC 103817 | RA_7895 | *O. nivara* | China | 63 |
|  | IRGC 104703 | RA_7912 | *O. nivara* | India | 64 |
|  | IRGC 105319 | RA_7917 | *O. nivara* | India | 65 |
|  | IRGC 105391 | RA_7919 | *O. nivara* | Thailand | 66 |
|  | IRGC 105343 | RA_7051 | *O. rufipogon* | India | 67 |
|  | IRGC 105843 | RA_7072 | *O. rufipogon* | Thailand | 68 |
|  | IRGC 81984 | RA_6989 | *O. rufipogon* | Laos | 69 |
|  | IRGC 82991 | RA_6999 | *O. rufipogon* | China | 70 |
|  | IRGC 80433 | RA_6970 | *O. rufipogon* | India | 71 |
|  | IRGC 106332 | RA_7103 | *O. rufipogon* | Cambodia | 72 |
|  | IRGC 106263 | RA_6787 | *O. rufipogon* | PapauNew guinea | 73 |
|  | IRGC 106273 | RA_7099 | *O. rufipogon* | PapauNew guinea | 74 |
|  | IRGC 105388 | RA_7053 | *O. rufipogon* | Thailand | 75 |
|  | IRGC 101979 | RA_7884 | *O. rufipogon* | India | 76 |
|  | IRGC 103305 | RA_6772 | *O. rufipogon* | Philippines | 77 |
|  | IRGC 105956 | RA_7083 | *O. rufipogon* | Indonesia | 78 |
|  | IRGC 104501 | RA_7031 | *O. rufipogon* | India | 79 |
|  | IRGC 106169 | RA_7098 | *O. rufipogon* | Vietnam | 80 |
|  | IRGC 105491 | RA_7057 | *O. rufipogon* | Myanmar | 81 |
|  | IRGC 106327 | RA_7102 | *O. rufipogon* | Cambodia | 82 |
|  | IRGC 100692 | RA_7868 | *O. rufipogon* | Taiwan | 83 |
|  | IRGC 105567 | RA_7059 | *O. rufipogon* | Indonesia | 84 |
|  | IRGC 105720 | RA_7065 | *O. rufipogon* | Cambodia | 85 |
|  | IRGC 82990 | RA_6998 | *O. rufipogon* | China | 86 |
|  | IRGC 100639 | RA_7864 | *O. rufipogon* | Taiwan | 87 |
|  | IRGC 106413 | RA_7109 | *O. rufipogon* | Vietnam | 88 |
|  | IRGC 101450 | RA_7880 | *O. rufipogon* | Taiwan | 89 |
|  | IRGC 81881 | RA_7118 | *O. rufipogon* | India | 90 |
|  | IRGC 106291 |  | *O. barthii* |  | 91 |
| CG10 | IRGC 96717 |  | *O. glaberrima* |  | 92 |
| CG17 | IRGC 86741 |  | *O. glaberrima* |  | 93 |
| CG20 | IRGC 96718 |  | *O. glaberrima* |  | 94 |
| IG10 | IRGC 103477 |  | *O. glaberrima* |  | 95 |
