# Supplementary material for: Exceptional lability of a genomic complex in rice and its close relatives revealed by interspecific and intraspecific comparison and population analysis
Source: BMC Genomics. 2011 Mar 8;12:142. doi: 10.1186/1471-2164-12-142 (PMC3060143; doi:10.1186/1471-2164-12-142)
Supplement: Additional file 3 — PCR verification of a segmental inversion present in O. glaberrima, but absent in O. sativa and O. nivara varieties. (A) Schematic primer design for amplification of inversion boundaries. (B) PCR products of primer pairs F1/R1 (panel b) and F2/R2 (panel c), and Waxy genes (panel a, control). Primers are shown in Additional file 4. Varieties are numbered according to their orders in Additional file 2. [file 1471-2164-12-142-S3.PPT]

## Slide 1
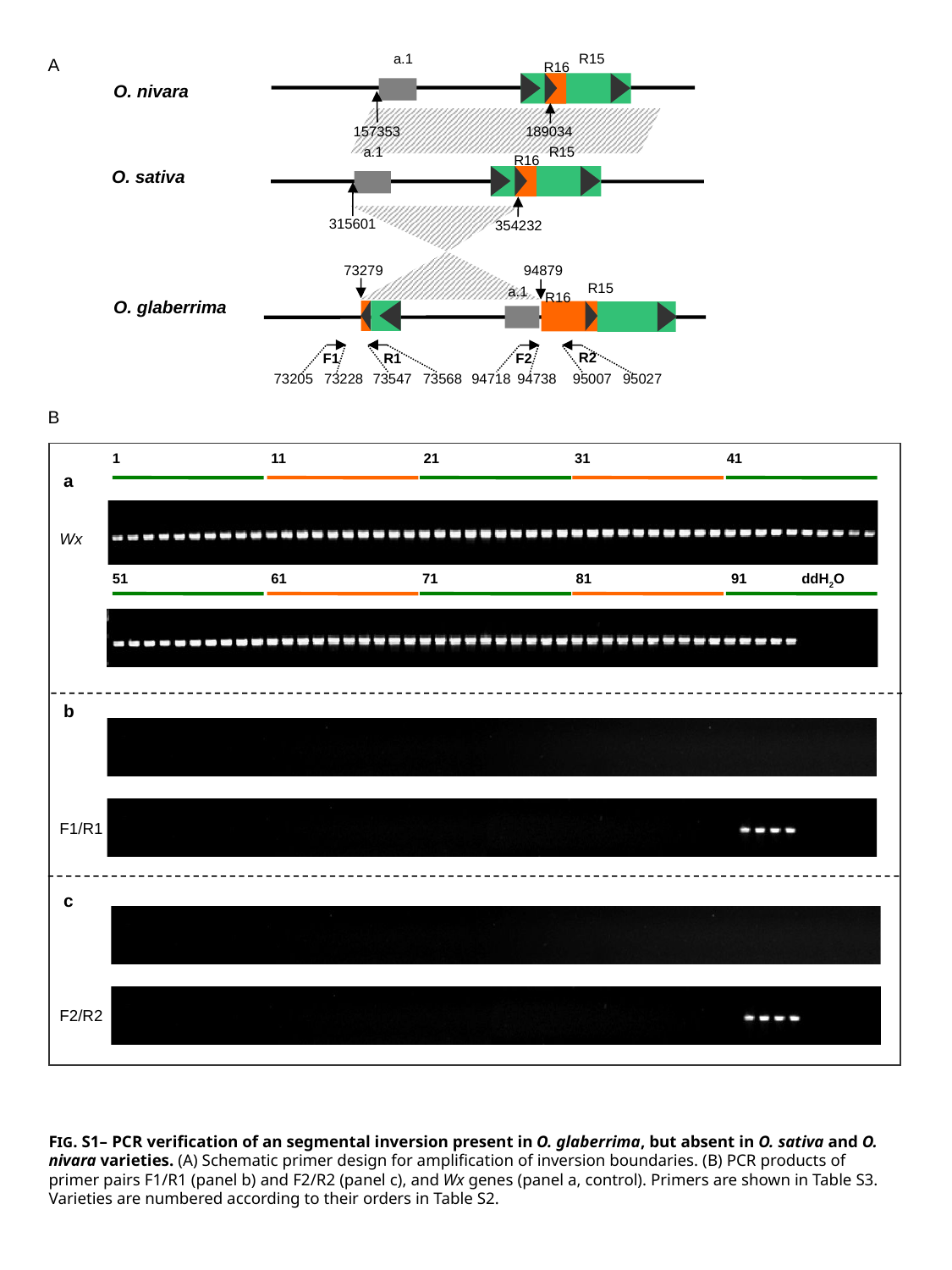

a.1
R15
A
R16
O. nivara
157353
189034
a.1
R15
R16
O. sativa
315601
354232
73279
94879
R15
a.1
R16
O. glaberrima
R2
F1
R1
F2
73228
73568
73205
73547
94738
95027
94718
95007
B
1
11
21
31
41
a
Wx
51
61
71
81
91
ddH2O
b
F1/R1
c
F2/R2
FIG. S1– PCR verification of an segmental inversion present in O. glaberrima, but absent in O. sativa and O. nivara varieties. (A) Schematic primer design for amplification of inversion boundaries. (B) PCR products of primer pairs F1/R1 (panel b) and F2/R2 (panel c), and Wx genes (panel a, control). Primers are shown in Table S3. Varieties are numbered according to their orders in Table S2.
