# Supplementary material for: Exceptional lability of a genomic complex in rice and its close relatives revealed by interspecific and intraspecific comparison and population analysis
Source: BMC Genomics. 2011 Mar 8;12:142. doi: 10.1186/1471-2164-12-142 (PMC3060143; doi:10.1186/1471-2164-12-142)
Supplement: Additional file 4 — Primers used in this study. Primers used to check the polymorphisms of LTR-retrotransposon insertions and the inversion in different species/subspecies. [file 1471-2164-12-142-S4.DOC]

| **Table S3. Primers used in this study** | | |
| --- | --- | --- |
| Primer sets | Forward primer sequences (5'->3') | Reverse primer sequences (5'->3') |
| Orp-RT6 | CTGTTTCGTGCCCACAATAGC | GCTGAACCGACAATACTGCTG |
| Orp-RT7 | TGGATACAACATCACATCTTTGA | GAGACATCGGACCCTACACG |
| Orp-RT9 | CTGGAAGAGCGACTGGTTCTAC | ACGCATTTTAGGCTTTTAGGAA |
| Orp-RT11 | CCACCACTCCTCCACTCCAT | AGGAAACCGATACCGTATTGGT |
| Orp-RT12 | GCAATGACCGAACTAACATATCTG | CCAATACGGTATCGGTTTCCT |
| Orp-RT14 | GAAGGAGAAGGTGGTGGAGG | GTCTTTCCGTCACCTCGGTA |
| Orp-RT15 | TCGGCTCAAATATAAATGATATGTC | TCTTGAGAATCTTCCCTAACTACC |
| Orp-RT19 | CTTCCGTTGTTTCCCGTTTG | GAGAGAAATGCTCGCTGCTG |
| Orp-RT21 | GCTCCGAAAAATCCGAGAA | GGATTTGGGAGAGGCTGATG |
| Orp-RT24 | TCTTCCATCACCTCCTTACGC | TCCGCTGCTGTTTTTCTTTTC |
| Orp-RT30 | CTACACCACCACCAAGAACTCC | CTTTTGGTTTGCCACTGTTTG |
| Orp-RT31 | CTGTGAAGATGGAGCCGTAGG | GTCTTTCCACCTGAACAGCAG |
| Orp-RT32 | CTGGTTGCCGATTAGGGTAG | GCGAATAAGATCGGCATGATG |
| Orp-inversion-L | TGATAACAAATTGTCTCACGAGTG | GCTGAACTAAAAGTGGCTGAGA |
| Orp-inversion-R | AGATAAGGCAGCGGAGTTAAG | TCTGAGAATGGAAGGGATGG |
