# Supplementary material for: Exceptional lability of a genomic complex in rice and its close relatives revealed by interspecific and intraspecific comparison and population analysis
Source: BMC Genomics. 2011 Mar 8;12:142. doi: 10.1186/1471-2164-12-142 (PMC3060143; doi:10.1186/1471-2164-12-142)
Supplement: Additional file 5 — Neighbor-Joining "Phylogeny" of the AA-genome varieties constructed using MEGA4 based on the presence/absence of a set of LTR-retrotransposons in individual varieties as illustrated in Figure 5. Varieties are numbered according to their orders shown in Figure 5 and Additional file 2. [file 1471-2164-12-142-S5.PPT]

## Slide 1
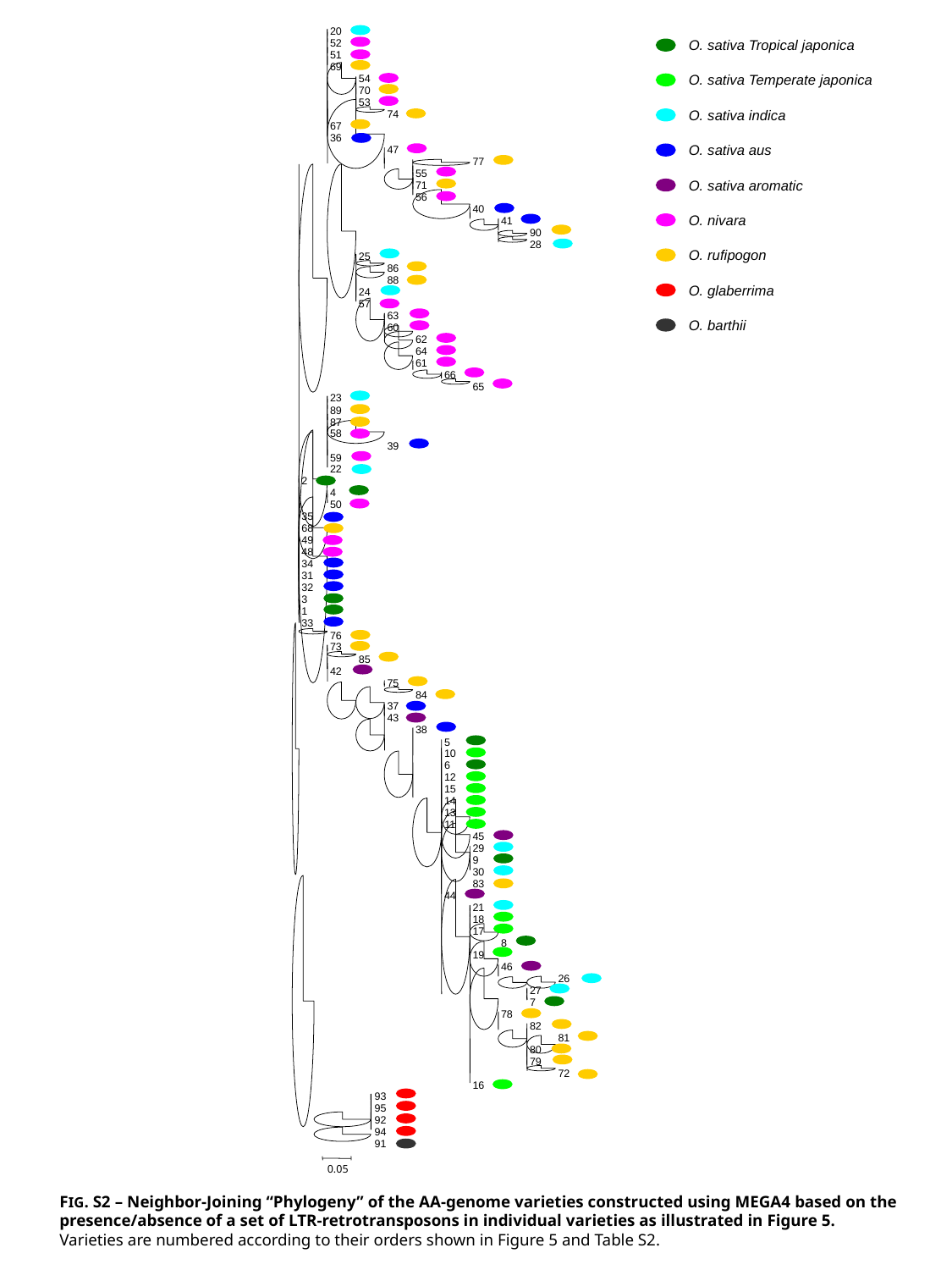

20
O. sativa Tropical japonica
 52
 51
 69
O. sativa Temperate japonica
 54
 70
 53
O. sativa indica
 74
 67
 36
O. sativa aus
 47
 77
 55
O. sativa aromatic
 71
 56
 40
O. nivara
 41
 90
 28
O. rufipogon
 25
 86
 88
O. glaberrima
 24
 57
 63
O. barthii
 60
 62
 64
 61
 66
 65
 23
 89
 87
 58
 39
 59
 22
 2
 4
 50
 35
 68
 49
 48
 34
 31
 32
 3
 1
 33
 76
 73
 85
 42
 75
 84
 37
 43
 38
 5
 10
 6
 12
 15
 14
 13
 11
 45
 29
 9
 30
 83
 44
 21
 18
 17
 8
 19
 46
 26
 27
 7
 78
 82
 81
 80
 79
 72
 16
 93
 95
 92
 94
 91
0.05
FIG. S2 – Neighbor-Joining “Phylogeny” of the AA-genome varieties constructed using MEGA4 based on the presence/absence of a set of LTR-retrotransposons in individual varieties as illustrated in Figure 5. Varieties are numbered according to their orders shown in Figure 5 and Table S2.
